# Efficacy of isolated femoral revision in cruciate‐retaining total knee arthroplasty instability: A comparative study

**DOI:** 10.1002/jeo2.70337

**Published:** 2025-08-20

**Authors:** Lars‐Rene Tuecking, Mats Tobias Wormit, Henning Windhagen, Max Ettinger, Peter Savov

**Affiliations:** ^1^ Department of Orthopaedic Surgery, Hannover Medical School Diakovere Annastift Hannover Germany; ^2^ Hannover Medical School Hannover Germany; ^3^ Department of Orthopaedic and Trauma Surgery, Pius Hospital Carl von Ossietzky University of Oldenburg Oldenburg Germany

**Keywords:** cruciate‐retaining, flexion instability, posterior‐stabilized, revision, total knee arthroplasty

## Abstract

**Purpose:**

This study compares clinical outcomes, implant survival rates and perioperative factors between isolated femoral total knee arthroplasty (TKA) revision (prTKA) and full TKA revision (frTKA) for flexion instability in cruciate‐retaining (CR) prostheses.

**Methods:**

This retrospective, controlled case series included 66 consecutive patients treated with either full TKA revision (*n* = 34) or isolated femoral TKA revision (*n* = 32) with flexion instability after CR TKA between 2015 and 2021. To ensure that the groups were uniformly comparable, only patients with one implant system (Triathlon, Stryker) were included. Preoperative demographic data and radiological parameters (e.g., quantification of anteroposterior instability and midflexion instability) were compared between the groups. Postoperative evaluation of implant survival and clinical outcome scores was performed with a minimum follow‐up of 2 years. Patient‐reported outcome measures (PROM) analysis included the Visual Analogue Scale, Kujala, Oxford Knee Score, Western Ontario and McMaster Universities Osteoarthritis Index, Forgotten Joint Score, University of California at Los Angeles Activity‐Level Scale and Knee Osteoarthritis Outcome scores. Statistical evaluations included unpaired, nonparametric *t*‐tests and Wilcoxon tests for nominal data. Implant survival analysis was conducted using Kaplan–Meier analysis and log‐rank test. Statistical significance was defined as a *p*‐value < 0.05.

**Results:**

No significant differences were found in the clinical outcomes between the prTKA and frTKA groups across various PROMs. Implant survival rates were comparable (96.9% for prTKA vs. 97.1% for frTKA). Compared to frTKA, prTKA resulted in significantly shorter hospital stays (*p* = 0.002), reduced operative time (*p* < 0.001), lower blood loss (*p* = 0.001) and a decreased inflammatory response (*p* < 0.001).

**Conclusions:**

Partial femoral TKA revision for flexion instability in cruciate‐retaining prostheses yielded clinical outcomes and implant survival rates comparable to full TKA revision in the short‐ to mid‐term follow‐up. These findings suggest that partial femoral revision may be a viable option for carefully selected patients with flexion instability, offering similar clinical efficacy and potential perioperative advantages over complete revision.

**Level of Evidence:**

Level III.

**Abbreviations**
a.p.: anteroposterior
BMI: body mass index
CCK: condylar constrained knee
CR: cruciate‐retaining
CRP: C‐reactive protein
FJS: Forgotten Joint Score
frTKA: full total knee arthroplasty revision
Hb: haemoglobin
HKA: hip‐knee‐ankle angle
KOOS: Knee Injury and Osteoarthritis Outcome Score
LDFA: lateral distal femoral angle
MPTA: medial proximal tibia angle
OKS: Oxford Knee Score
PCL: posterior cruciate ligament
PROM: patient‐reported outcome measure
prTKA: partial total knee arthroplasty revision
PS: posterior‐stabilized
TKA: total knee arthroplasty
UCLA: University of California at Los Angeles Activity‐Level Scale
VAS: Visual Analogue Scale
WOMAC: Western Ontario and McMaster Universities Osteoarthritis Index

## INTRODUCTION

Total knee arthroplasty (TKA) has emerged as a highly effective and widely adopted surgical intervention for treating end‐stage knee osteoarthritis. As the population ages and the prevalence of osteoarthritis increases, the number of primary TKA procedures performed annually continues to rise steadily [12, 13]. Consequently, a proportional increase in the incidence of revision surgeries is anticipated [12, 13], presenting a growing challenge for orthopaedic surgeons and healthcare systems [4].

Revision TKA procedures are inherently more complex and demanding than primary surgeries, often resulting in less favourable clinical outcomes [8, 23] and higher rates of re‐revision [23]. These challenges underscore the critical importance of addressing the underlying causes of failure effectively and developing innovative approaches to improve the longevity and success of both primary and revision TKA procedures. The complexity of revision surgeries stems from various factors, including altered anatomy, potential bone loss and the need to remove well‐fixed implants with the smallest possible damage to the surrounding soft tissues and attached bone [22].

Among the multitude of reasons for TKA revision, TKA instability still remains one of the most common reasons [18]. Particularly noteworthy is flexion instability, which can manifest itself as midflexion or sagittal instability [20, 21, 25]. Sagittal instability can arise from various factors, including secondary degeneration of the posterior cruciate ligament (PCL) over time or iatrogenic injury to the PCL during the primary procedure [11]. On the other hand, mid‐flexion instability is associated with malpositioning or incorrect prosthesis sizing of the femoral component and resulting reduction of posterior condylar offset [6].

Contrary to other reasons for TKA revision, instability can rarely be corrected by correcting the tibial component or by isolated exchange of the polyethylene liner [2, 5, 26, 29]. Instead, in these cases, it is mostly necessary to address the femoral component to be able to adjust the constraint level of the implant. In flexion instability, compensation for the PCL insufficiency in cruciate‐retaining (CR) TKA with an increase of the constraint level [9] or correction of the posterior condylar offset is often necessary [25].

In cases of tibial loosening or isolated malpositioning of the tibial component, it has been demonstrated that partial revision of only the tibial component yields comparable results and implant survival rates to complete TKA revision [3, 14, 17, 28]. Advantages of this approach include reduced operative time, lower invasiveness and shorter hospital stays [14, 28].

Currently, fewer studies exist on partial revision of the femoral component, particularly in cases of flexion instability. Given the anticipated increase in revision procedures in TKA surgery, it is necessary to develop and evaluate new methods to reduce invasiveness and improve patient outcomes. The current data on isolated femoral revisions is insufficient. A recent study demonstrated comparable clinical outcomes after isolated femoral revision compared to complete revision, in which numerous indications and implant types were compared [24].

Therefore, the aim of this study is to compare partial femoral TKA revision with complete TKA revision in a defined indication using a specific prosthesis type from a single manufacturer.

The present study hypothesizes that in cases of flexion instability with CR prostheses, where the tibial component's position and condition are satisfactory, an isolated femoral TKA revision to a posterior‐stabilized (PS) prosthesis can yield clinical outcomes and implant survival rates comparable to those of full TKA revisions in short‐ to mid‐term follow‐up periods.

## METHODS

### Study design

This is a single‐centre retrospective study conducted on patients with flexion instability after primary TKA (Triathlon® CR system) who underwent partial (femoral) or full TKA revision from 2015 to 2021 with the same single implant system (Triathlon®, Stryker) at a tertiary arthroplasty centre. Patients were assigned to one of two groups: partial femoral revision TKA (prTKA) or full revision TKA (frTKA). All data were collected retrospectively via self‐administered patient‐reported outcome measurement (PROM) and clinical course questionnaire surveys. Informed consent was obtained from each participating patient. Perioperative data were extracted from the clinical information system of the study hospital. Ethical approval for this study was obtained from the local Ethics Committee (EK‐Nr.:8437).

### Inclusion and exclusion criteria

All patients revised for flexion instability with the implanted Triathlon® CR system from 2015 to 2021, undergoing revision TKA surgery using the Triathlon® system, were included. The exclusion criteria were different implant systems, different causes of revision (infection, arthrofibrosis and loosening) and mobility impairment due to secondary diagnoses (e.g., neurological pathologies).

### Operative technique

The feasibility of partial TKA depends on the preoperative diagnosis and the cause of flexion instability. Patients exhibiting flexion instability (e.g., insufficiency of the PCL accompanied by pathological anteroposterior [a.p.] translation in a.p. stress images [19]), with an implanted tibia showing no indications of an elevated posterior tibial slope (>3°), and which also demonstrated suitable coronal alignment of the tibial component (medial proximal tibial angle [MPTA], 87°–90°), were deemed appropriate candidates for partial revision. It was necessary to ensure this in order to combine a femoral PS component with the retained tibial component. The primary goal was always to perform a partial femoral revision and to check for criteria that exclude such a partial revision (see above, e.g., high tibial slope, valgus malalignment of the tibia). All patients of the prTKA group received a cemented Triathlon® PS femur implant with PS fixed bearing onlay; implant selection in the frTKA was mainly the condylar constrained version of this implant (Triathlon® TS with TS onlay, 31/34 [91.2%]), with 3/34 (8.8%) Triathlon® PS femur implant with PS fixed bearing onlay.

### Outcome measures and data collection

**Figure 1:**
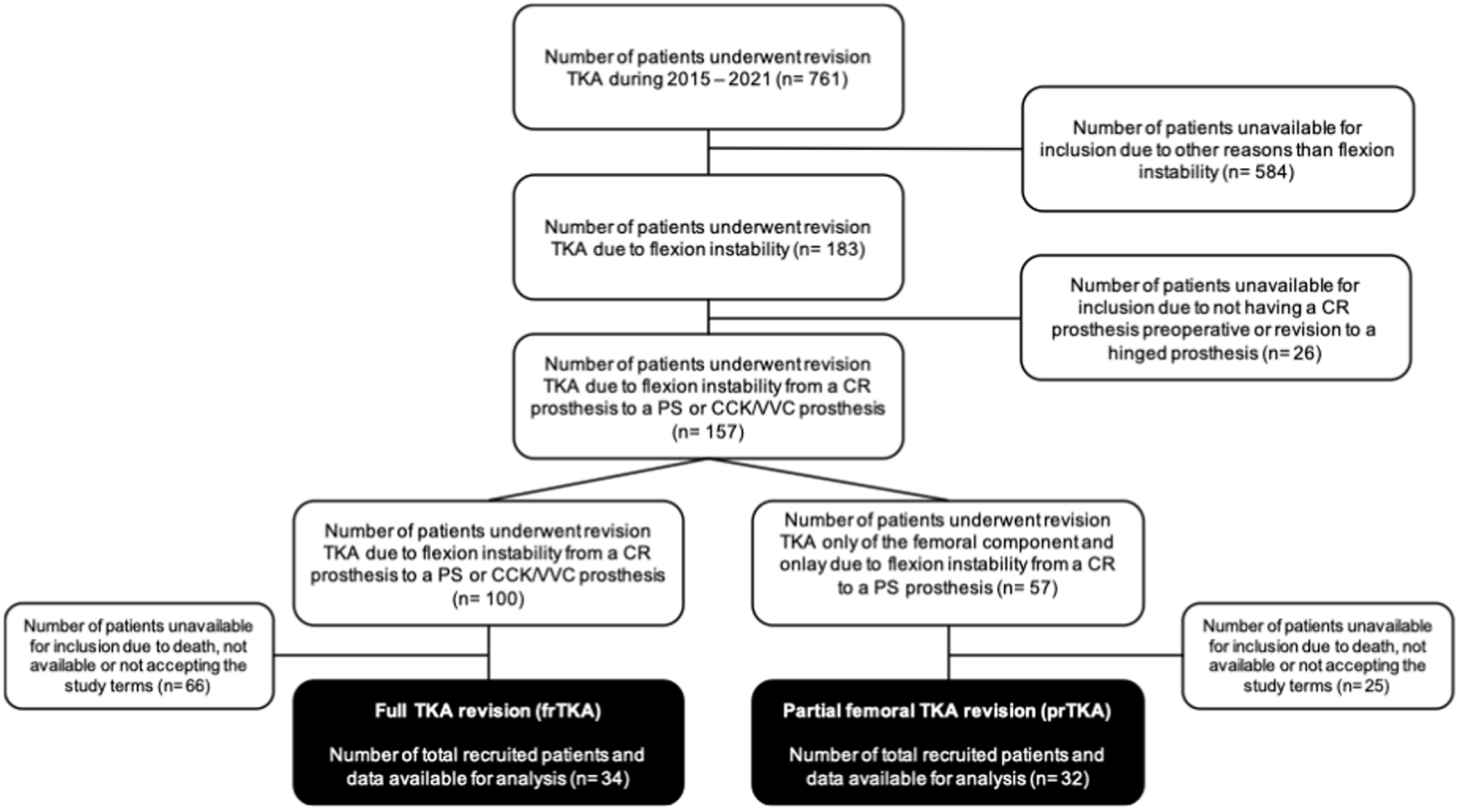
Group allocation of patients. CCK, condylar constrained knee; CR, cruciate‐retaining; PS, posterior‐stabilized; TKA, total knee arthroplasty.

All consecutive patients who underwent surgery between 2015 and 2021 with the above inclusion criteria were recorded, resulting in a minimum follow‐up of two years. Demographic and perioperative data were extracted from the institutional clinical information system. Subsequently, the patients were contacted by mail, and written informed consent was obtained. All PROMs described below were collected via self‐administered questionnaires, and a survey on implant survival was conducted. All patients without a postal response were contacted by telephone and were reminded of their possible participation. Patients who could not be contacted by post or telephone, or with missing or rejected informed consent, were excluded (see Figure 1). PROMs for postoperative clinical outcome included Forgotten Joint Score (FJS), Oxford Knee Score (OKS), Knee Injury and Osteoarthritis Outcome Score (KOOS), Kujala Score, Western Ontario and McMaster Universities Osteoarthritis Score (WOMAC), Visual Analogue Pain Scale (VAS), University of California at Los Angeles Activity‐Level Scale (UCLA). Demographic and perioperative data at the time included age, sex, body mass index (BMI), implant specifications (constraint level, modular components, onlay type and onlay height in mm), length of stay in days, difference in pre‐ to postoperative (Day 1 postoperative), haemoglobin level (Hb) in g/dL, incision time in min and implant costs in EUR. The radiological examination included long knee radiographs in two planes with full‐leg radiographs, as well as a.p. and midflexion varus‐valgus stress views preoperatively. Postoperative radiographic examination included two‐plane long‐knee radiographs with full‐leg radiographs. Radiological measurements included the hip‐knee‐ankle angle, lateral distal femoral angle (LDFA), MPTA, posterior tibial slope and posterior condylar offset ratio.

### Statistical analysis

Statistical analyses were performed using the IBM SPSS Statistics software, version 28 (IBM Corp.). Descriptive statistics were reported as means and standard deviations for continuous variables and frequencies and percentages for categorical variables. Further statistical evaluations included unpaired, nonparametric *t*‐tests and Wilcoxon tests for nominal data. Clinical outcome scores were only compared between the revision‐free patients. Kaplan–Meier survival analysis was used to estimate implant survival using log‐rank testing for group comparisons. Failure was defined as revision surgery for any cause. Additionally, a separate analysis was performed to assess infection‐free survival, with failure defined as revision surgery other than infection. Patients were censored if they had not undergone revision at the time of last follow‐up or had died without undergoing revision. Statistical significance was set at *p* < 0.05.

## RESULTS

A total of 66 patients were included in both groups, with 32 patients in the prTKA group and 34 patients in the frTKA group (Figure 1), with a mean follow‐up time of 54.1 ± 26.1 months. Preoperative demographic data showed no differences between the two groups (*p* > 0.05, Table 1), with the exception of a slight difference found preoperatively in the comparison of the full leg axes with a slightly more valgus leg axis in the frTKA group compared to the prTKA group (179.2 ± 2.7 vs. 181.6 ± 4.9, *p* = 0.02).

**Table 1:** Demographic data of groups.

	Partial TKA revision	Full TKA revision	*p* value
Age (years)	63.9 ± 10.8	65.1 ± 11.8	0.652
Sex (female, in %)	23, 76.7%	22, 64.7%	0.296
BMI (kg/m^2^)	30.4 ± 5.1	30.4 ± 5.4	0.992
HKA in °			
Preoperative	179.2 ± 2.7	181.6 ± 4.9	0.020
Postoperative	179.9 ± 2.7	179.5 ± 2.8	0.532
mMPTA in °			
Preoperative	89.8 ± 1.6	89.2 ± 2.2	0.158
Postoperative	‐‐‐	88.8 ± 1.8	0.014
mLDFA in °			
Preoperative	89.4 ± 2.6	89.7 ± 3.3	0.699
Postoperative	88.7 ± 2.4	88.7 ± 2.1	0.952

*Note*: Mean ± standard deviation.

Abbreviations: BMI, body mass index; HKA, hip‐knee‐ankle angle; mLDFA, mechanical lateral distal femoral angle; mMPTA, mechanical medial proximal tibia angle; TKA, total knee arthroplasty.

### Clinical outcome parameters

The results of the PROMs are listed in Table 2. Clinically, there was no significant difference across PROMs (all *p* > 0.05) between the two groups, with a minimum follow‐up of 2 years. Minimal benefits were observed for all scores in the prTKA group (Table 2).

**Table 2:** Postoperative patient‐reported outcome measurements.

	Partial TKA revision	Full TKA revision	*p* value
VAS rest			
Postoperative	2.6 ± 2.9	2.4 ± 2.2	0.735
VAS load			
Postoperative	4.7 ± 3	5.1 ± 2.7	0.571
OKS			
Postoperative	30.2 ± 9.8	28.2 ± 10.2	0.440
WOMAC			
Postoperative	34.5 ± 22.7	36.7 ± 21.4	0.693
FJS			
Postoperative	27.5 ± 19.9	23.5 ± 26.3	0.150
UCLA			
Postoperative	5.1 ± 1.9	5 ± 1.6	0.816
PHQ‐9‐D			
Postoperative	6.6 ± 6.2	5.9 ± 5.7	0.925
Kujala			
Postoperative	55.2 ± 16.7	51.3 ± 20.2	0.413
KOOS pain			
Postoperative	60.8 ± 21.6	55.3 ± 19.9	0.292
KOOS symptoms			
Postoperative	67 ± 14.8	63 ± 19.4	0.374
KOOS ADL			
Postoperative	64.6 ± 22.2	60.1 ± 21.5	0.420
KOOS sports			
Postoperative	26.2 ± 21.2	24.6 ± 24.3	0.780
KOOS QOL			
Postoperative	36.3 ± 21.7	34.7 ± 21	0.930

*Note*: Mean ± standard deviation.

Abbreviations: ADL, activities of daily life; FJS, Forgotten Joint Score; KOOS, Knee Osteoarthritis Outcome Score; OKS, Oxford Knee Score; PHQ‐9‐D, Patient Health Questionnaire‐9; QOL, quality of life; TKA, total knee arthroplasty; UCLA, University of California at Los Angeles Activity‐Level Scale; VAS, Visual Analogue Scale; WOMAC, Western Ontario and McMaster Universities Osteoarthritis Index.

### Perioperative outcome parameters

The perioperative outcome parameters are summarized in Table 3. PrTKA showed significantly lower length of stay (7.2 ± 2.5 vs. 11.1 ± 10.3, *p* = 0.002), lower overall incision time (75.7 ± 27.3 vs. 108.7 ± 31.1, *p* < 0.001), lower delta value of Hb (pre‐ to first day postoperative, −2.2 ± 1 vs. −3.3 ± 1.3, *p* = 0.003) and lower C‐reactive protein (CRP) values (2.5 ± 3.2 vs. 5.5 ± 6.1, *p* = 0.001 [Day 1 postoperative]; 6.3 ± 4.1 vs. 14.2 ± 10.3, *p* < 0.001 [Day 3 postoperative]) when compared to frTKA.

**Table 3:** Perioperative outcome parameters.

	Partial TKA revision	Full TKA revision	*p* value
Length of stay (days)	7.19 ± 2.5	11.14 ± 10.3	0.002
Incision time (min)	75.7 ± 27.3	108.7 ± 31.1	<0.001
Haemoglobin (g/dL)			
preoperative	13.9 ± 1.2	13.8 ± 1	0.697
Day 1 postoperative	11.7 ± 1.1	10.5 ± 1.3	0.001
Delta value	−2.2 ± 1	−3.3 ± 1.3	0.003
CRP (mg/dL)			
Preoperative	0.36 ± 0.3	0.48 ± 0.6	0.323
Day 1 postoperative	2.5 ± 3.2	5.5 ± 6.1	0.001
Day 3 postoperative	6.3 ± 4.1	14.2 ± 10.3	<0.001
Day 5 postoperative	6.9 ± 6.9	11.1 ± 9.1	0.054

*Note*: Mean ± standard deviation.

Abbreviations: CRP, C‐reactive protein; TKA, total knee arthroplasty.

### Implant survival analysis

**Figure 2:**
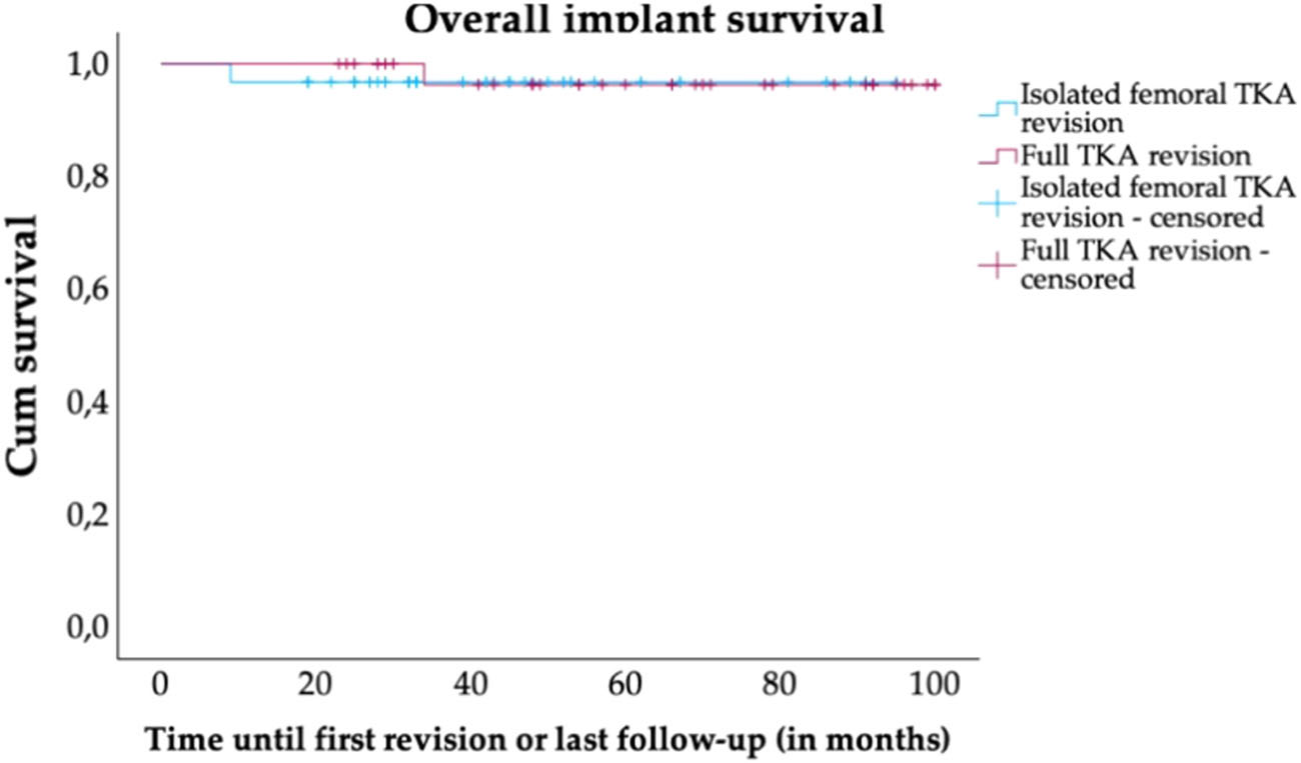
Overall implant survival. Kaplan–Meier survival curves for partial TKA revision and full TKA revision groups. Censored patients are indicated by tick marks, representing patients who did not undergo revision at the follow‐up timepoint or died without undergoing revision. TKA, total knee arthroplasty.

**Figure 3:**
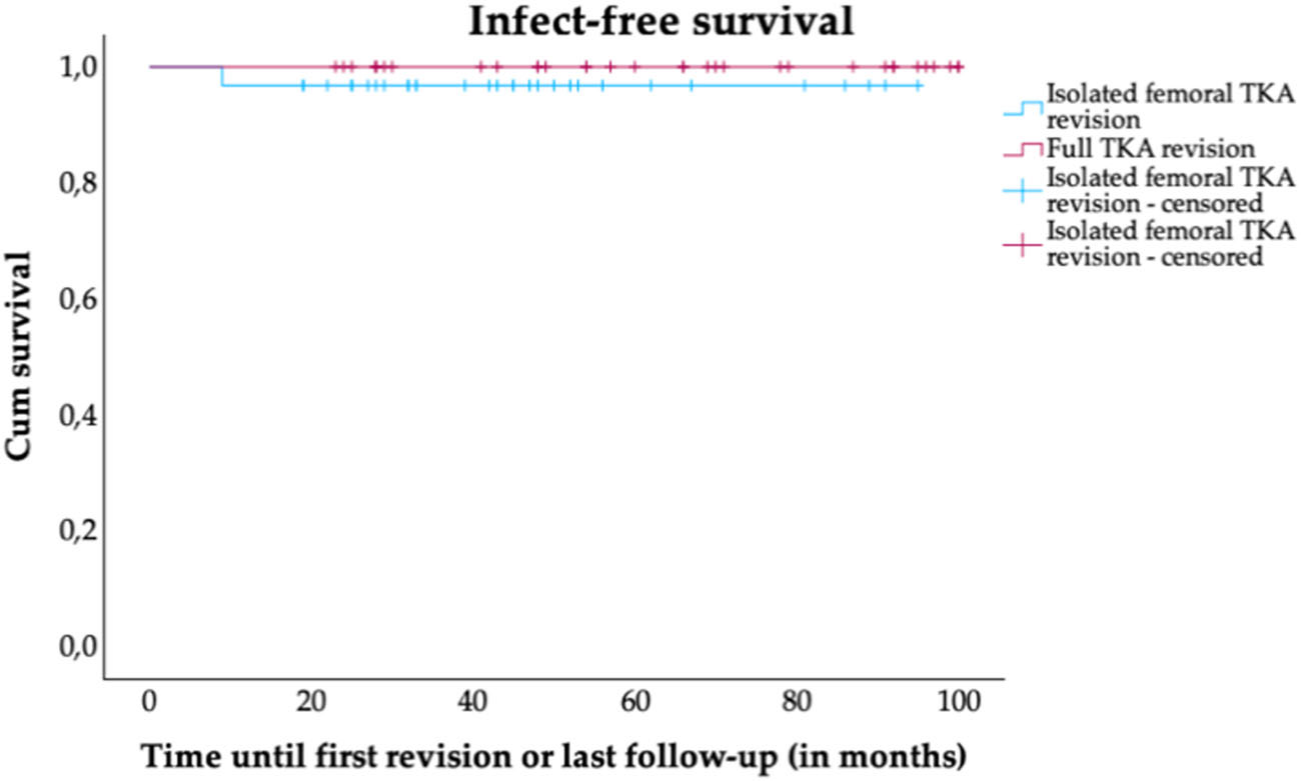
Infect‐free implant survival. Kaplan–Meier survival curves for partial TKA revision and full TKA revision groups. Censored patients are indicated by tick marks, representing patients who did not undergo revision at the follow‐up timepoint or died without undergoing revision. TKA, total knee arthroplasty.

The survival analysis is shown in Figures 2, 3. Kaplan–Meier overall survival analysis revealed no significant difference between prTKA and frTKA (*p* = 0.852). The estimated 5‐year survival rates were 97.1% (95% confidence interval [CI]: 92.9%–100%) for frTKA and 96.8% (95% CI: 86.9%–97.6%) for prTKA. Kaplan–Meier infect‐free survival analysis revealed no significant difference between prTKA and frTKA (*p* = 0.295) with estimated 5‐year survival rates of 100% for frTKA and 96.8% (95% CI: 86.9%–97.6%) for prTKA. Each group showed one revision in the follow‐up period (frTKA: T84.5, periprosthetic joint infection; prTKA: T84.05, recurrence of TKA instability).

## DISCUSSION

As the key findings of this study, short‐ to mid‐term follow‐up revealed no significant differences in clinical outcomes between prTKA and frTKA for flexion instability in CR prostheses, suggesting both approaches achieve comparable functional recovery. Although prTKA demonstrated slightly better scores across PROMs, the differences did not exceed the established minimal clinically important differences (MCID) for the OKS (MCID ~ 4 points) and WOMAC (MCID ~ 10 points). Additionally, there was no observed difference in implant survival between the two techniques within this timeframe, suggesting that both revision approaches yielded comparable clinical outcomes and implant longevity. However, the study revealed a significant reduction in operative time, shorter hospital stay, lower blood loss and reduced inflammatory response in the partial femoral revision group. This suggests that while prTKA may offer perioperative benefits, the clinical impact on patient‐reported outcomes remains marginal.

### Clinical outcome

A comparable clinical outcome between the two revision techniques has also been reported in most studies comparing full and partial revisions [1, 3, 7, 14, 16, 17, 24]. However, the overall body of evidence on this topic is sparse, and the heterogeneity in study design, particularly concerning the included revision indications, implant systems and surgical techniques (both tibial and femoral revisions), prevents direct comparisons across studies. Some authors have described inferior KSS outcome scores after partial revisions for TKA instability [7], especially when using PS implants [24]. In contrast, our findings did not corroborate these results, as we observed comparable postoperative scores using PS implants for partial revision compared with condylar constrained knee (CCK) implants in full TKA revision. In the study by Fehring et al. [7], the comparison between the two revision techniques was based on only 11 partial revisions for TKA instability, the majority of which were tibial revisions. It should be noted that partial tibial revision in cases of TKA instability is significantly more complex because changes in tibial component alignment can affect both the extension and flexion gaps. In cases where the extension gap is balanced but flexion instability is present (e.g., flexion instability), addressing the pathology solely through tibial revision is highly complex and may not always be feasible. This underscores the importance of conducting comparative analyses with a single defined indication for revision surgery by using a closed prosthesis system.

### Implant survival

A crucial metric for comparing different surgical techniques in revision arthroplasty is implant survival, which has already been shown to be comparable between partial and full TKA revision [7, 16, 24]. We were able to confirm these findings for partial TKA revisions in CR prostheses with a short‐ to mid‐term follow‐up in the context of TKA instability. With accurate preoperative diagnosis and proper assessment of instability, as well as precise evaluation of the tibial component's positioning, partial femoral revisions can achieve similar implant survival rates to full revisions for CR‐type TKA instability. Considering the factors outlined above, patient selection and appropriate indications are likely critical in achieving these outcomes. The risk of re‐revision due to persistent instability following conversion to a PS implant remains a subject of ongoing discussion. The only revision in the partial TKA revision group was persistent instability, which required further revision of the CCK prosthesis. Similarly, Shichman et al. [24] reported a 16‐fold increased risk of re‐revision for PS implants and a two‐fold increased risk compared with hinge prosthesis types in cases of TKA instability. A registry analysis by Lewis et al. [15] found a 30% increased likelihood of revision for partial revisions in TKA instability, with the lowest re‐revision rates observed in hinge prostheses. Two main factors should be considered in this context. First, it is important to note that the inclusion of various implant systems, as well as the additional inclusion of partial tibial revisions [24], may influence the outcomes after partial revisions in cases of TKA instability. The inclusion of partial tibial revisions involves greater technical complexity, as previously mentioned, and may influence the postoperative outcomes of partial TKA revision. In contrast, femoral reconstruction with distal and posterior augments allows for more flexible adjustments to prosthetic stability by selectively modifying flexion or extension gaps. Second, both groups in this study demonstrated excellent survival rates in short‐ to mid‐term follow‐up. Statistically, in contrast to the previously cited studies, no significant difference was observed between the use of PS or CCK prostheses or between partial and full TKA revisions. This study successfully demonstrated the clinical efficacy of partial revisions using PS prostheses in carefully selected cases.

### Perioperative factors

A closer analysis of perioperative factors revealed significant differences between the two surgical approaches, consistent with the findings of comparable studies [17, 28]. The significant reductions in hospital stay (prTKA: 7.19 ± 2.5 days vs. frTKA: 11.14 ± 10.3 days, *p* = 0.002) and operative time (prTKA: 75.7 ± 27.3 min vs. frTKA: 108.7 ± 31.1 min, *p* < 0.001) suggest potential cost savings. The partial revision method also resulted in a lower inflammatory response, as indicated by reduced markers of inflammation and less intraoperative blood loss. These perioperative advantages are important from a healthcare resource and cost perspective, and could contribute to faster recovery and reduced morbidity in the immediate postoperative period. Future cost‐analysis studies are needed to confirm the economic advantages of prTKA.

### Limitations

The main limitation of this study is its retrospective design, which inherently affects the reliability of the data collected and the validity of the conclusions drawn. The relatively small sample size (prTKA: 32, frTKA: 34) limits statistical power, increasing the risk of a Type II error. A larger, prospective cohort is needed to validate these findings. Nevertheless, both groups were comparable in terms of their demographic characteristics. In addition, there was a higher rate of patients who could not be reached or who refused to participate in the study in the frTKA group. This could influence the results, as there may be different revision rates or postoperative clinical scores in the non‐participating patient groups. To ensure optimal comparability between partial and full TKA revisions, only patients with a specific diagnosis of instability, identical revision implant and the same constraint level were included. Despite the retrospective study design, the highly specific inclusion criteria resulted in a limited sample size. However, this also distinguishes the present study from previously published studies on partial TKA revisions, some of which have included larger overall sample sizes. When considering subgroup analyses, such as those focusing on TKA instability, the number of cases is often smaller than that in the current study. Further studies with larger sample sizes and prospective designs are necessary to confirm these findings. Additionally, longer‐term follow‐up periods would be valuable and informative for comparing both revision techniques, although the primary factor for revision for these study groups, instability, tends to manifest clinically within the early postoperative period [10, 27]. Finally, the exclusive use of a single implant system (Triathlon, Stryker) ensures homogeneity in prosthetic design and surgical technique. However, this limits the generalizability of our findings to other implant systems, and future studies should explore whether similar outcomes are observed across different manufacturers.

## CONCLUSION

Comparable clinical outcome scores and implant survival were found for isolated femoral TKA revision compared with full TKA revision. In adequately chosen patients, isolated femoral TKA revision may be a possible solution to avoid full TKA revision, reducing the utilization of healthcare resources and perioperative morbidity of patients.

## AUTHOR CONTRIBUTIONS

*Conceptualization*: Lars‐Rene Tuecking, Max Ettinger and Peter Savov. *Data curation*: Mats Tobias Wormit. *Formal analysis*: Lars‐Rene Tuecking and Peter Savov. *Investigation*: Mats Tobias Wormit and Lars‐Rene Tuecking. *Methodology*: Lars‐Rene Tuecking, Peter Savov, Henning Windhagen and Max Ettinger. *Project administration*: Lars‐Rene Tuecking, Henning Windhagen, Peter Savov and Max Ettinger. *Supervision*: Max Ettinger and Henning Windhagen. *Visualization*: Mats Tobias Wormit. *Writing—original draft*: Lars‐Rene Tuecking and Peter Savov. *Writing—review and editing*: Max Ettinger, Henning Windhagen and Mats Tobias Wormit.

## CONFLICT OF INTEREST STATEMENT

M.E., L.‐R.T., P.S. and H.W. have been paid educational consultancy fees from Stryker, USA. The remaining author declares no conflicts of interest.

## ETHICS STATEMENT

This study was approved by the local Ethics Committee (EK‐Nr. 8437). Written informed consent was received from every included patient.

## Data Availability

The data that support the findings of this study are available from the corresponding author upon reasonable request.
